# Fabrication, Structure, and Mechanical and Ultrasonic Properties of Medical Ti6Al4V Alloys Part II: Relationship between Microstructure and Mechanical Properties and Ultrasonic Properties of Ultrasonic Scalpel

**DOI:** 10.3390/ma13020284

**Published:** 2020-01-08

**Authors:** Zheyu He, Hao He, Jia Lou, Yimin Li, Dongyang Li, Yongzhi Chen, Shaojun Liu

**Affiliations:** 1 State Key Laboratory for Powder Metallurgy, Central South University, Changsha 410083, China; holmes416@csu.edu.cn (Z.H.); liyimin333@163.com (Y.L.); dongyangl@csu.edu.cn (D.L.); csuchen@csu.edu.cn (Y.C.); 2 Research Center for Materials Science and Engineering, Guangxi University of Science and Technology, Liuzhou 545006, China; 3 School of Materials Science and Engineering, Xiangtan University, Xiangtan 411105, China; lou3166@xtu.edu.cn

**Keywords:** medical Ti6Al4V alloys, microstructure, elastic modulus, ultrasonic properties

## Abstract

In this study, the ultrasonic resonance parameters of Ti6Al4V alloys under different heat treatments are measured by an impedance analyzer. The amplitude of the specimens is measured experimentally by means of optical microscope and image analysis software. These results show that the ultrasonic properties of Ti6Al4V alloys are closely related to β phase content and elastic modulus of the alloys. The highest volume fraction of the β phase appears in the specimen treated by solid solution treatment at 960 °C is 40.2%. These alloys present the lowest average elastic modulus (~99.69 GPa) and the minimum resonant frequency (55.06 kHz) and the highest average amplitude (21.48 µm) when the testing sample length is 41.25 mm. These findings can be used to guide the design of medical Ti6Al4V alloys for ultrasonic scalpels.

## 1. Introduction

Ti6Al4V alloy is a two-phase (α + β) alloy widely used in the biomedical field owing to its excellent biocompatibility [1]. Especially in ultrasonic surgery, the Ti6Al4V alloy is widely used for ultrasonic scalpels, which require not only favorable biocompatibility, but also excellent mechanical and ultrasonic properties [2]. Most of the current investigations focus on the effect of ultrasonic processing on the wear and fatigue behavior of this Ti6Al4V alloy [3,4,5], as well as the effect of microstructures on the ultrasonic wave velocity and attenuation coefficient of Titanium alloys [6,7]. The influence of heat treatment on the mechanical properties of Ti6Al4V alloys have been described in detail in the first paper of a series of papers focusing on the fabrication, structure and mechanical and ultrasonic properties of medical Ti6Al4V alloys.

However, we would like to mention that the resonant frequency and amplitude of ultrasonic scalpels are considered as the two most important physical parameters that affect the tissue fragmentation and coagulation in ultrasonic surgery [8]. Zainon [9] found that when the mechanical vibration of the particle acceleration of 5 × 10^4^ × g (g is gravitational acceleration, g ≈ 10 m/s^2^) acts on the living biological tissue, the affected part can be quickly cut without hurting the organization around it. 

For ultrasonic scalpels with resonant frequency of 55 kHz, the amplitude must reach 16 µm to effectively cut soft tissue. For ultrasonic scalpels with resonant frequency of 20 kHz, the amplitude must be at least 32 µm [10,11]. 

It has been shown [12,13] that it is necessary to design the scalpels with a complex shape that can be fabricated by high precision machining to obtain a large amplitude. For example, for the ultrasonic cutting and coagulation Ti6Al4V alloy scalpels by Ethicon Endo-Surgery Inc., the resonant frequency is 55.5 kHz. The maximum amplitude ~80 µm of the scalpels can be satisfied by shape design, including the scissors and spherical solidification rod [12]. In contrast, for an ultrasonic scalpel with bendable waveguide made from NiTi wire with a diameter of 1 mm and length of 303 mm, the operating frequency and the maximum amplitude are 23.5 kHz and 98 µm, respectively [13]. However, the studies on the effect of microstructure on the ultrasonic resonant frequency and the amplitude of titanium alloys are relatively few. It is well known that titanium alloys have poor thermal conductivity and a high friction coefficient which make them very difficult to machine [14]. Therefore, it is more effective by adjusting the microstructure of the titanium alloys rather than the complex shape design to improve the ultrasonic properties of titanium alloys. It is clear it is of significant importance to clarify the relationship between the microstructure (such as phase content, size, etc.) and the ultrasonic characteristics (including ultrasonic resonance frequency and amplitude) and the mechanical properties of Ti6Al4V alloys.

This paper aims to address the factors that significantly influence the ultrasonic properties of the Ti6Al4V alloy and establish the relationship between the microstructure and the ultrasonic resonance frequency and the amplitude of Ti6Al4V alloys for ultrasonic scalpels.

## 2. Experiments

**Figure 1:**
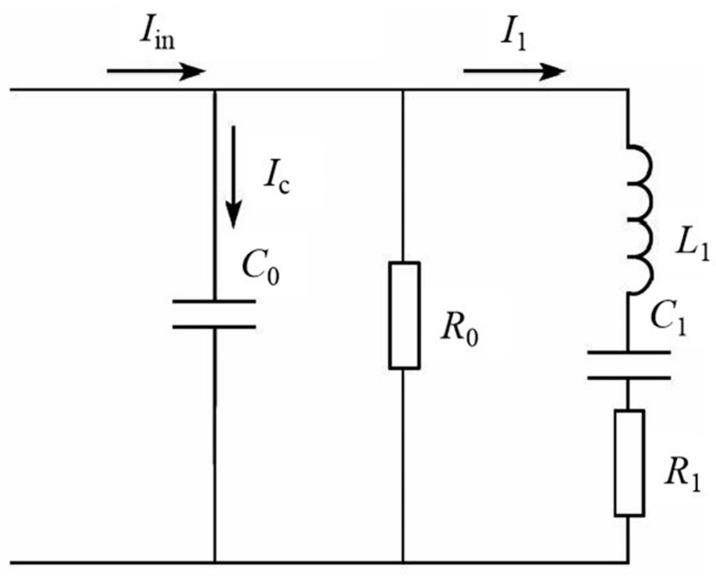
Equivalent circuit diagram of the ultrasonic vibrator.

In the present paper, the ultrasonic properties including the ultrasonic resonant parameter (URP) and amplitude were tested. For the URP testing, Ti6Al4V alloys with various microstructures tailored by different heat treatments were processed into cylindrical samples with a diameter of 4.5 mm and length of 41–50 mm (no special shape design to increase amplitude). The PV520A impedance analyzer (Funsonic Ultrasonic Technology, Hangzhou, China) was used to test the resonant frequency (*Fs*), dynamic resistance (*R*_1_) and mechanical quality factor (*Qm*) of the Ti6Al4V ultrasonic vibrator, which consisted of an ultrasonic transducer and Ti6Al4V alloy specimens. The equivalent circuit diagram [15] of the ultrasonic vibrator is shown in Figure 1. *L*_1_, *C*_1_ and *R*_1_ are connected in series to form a dynamic branch of the vibration system. *R*_1_ is the dynamic equivalent resistance of the ultrasonic vibrator, *L*_1_ is the dynamic equivalent inductance and *C*_1_ is the dynamic equivalent capacitance. *R*_0_ and *C*_0_ form a parallel static branch of the vibration system. To guarantee the veracity and reliability of experimental results, three samples were used in each test condition.

**Figure 2:**
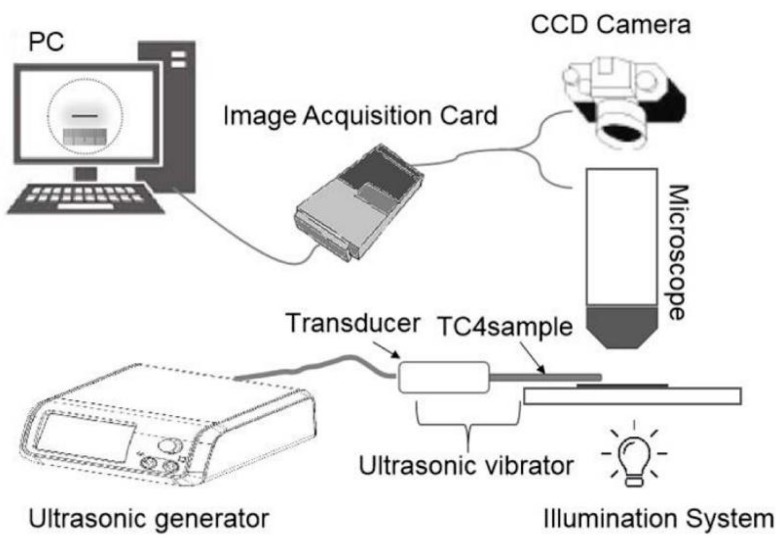
The schematic diagram of the ultrasonic amplitude measurement of the Ti6Al4V vibrator.

The ultrasonic generator used in the experiment is Johnson & Johnson’s GEN04 (Somerville, NJ, USA), with frequency in a range of 55.5 ± 1 kHz. Therefore, it is necessary to adjust the sample length so that the *Fs* of the Ti6Al4V alloys fall into this frequency range. For samples that match the frequency of the ultrasonic generator, the amplitude was observed by an optical microscope and recorded by a charge-coupled device camera (Suiou Instrument, Shanghai, China). The video of ultrasonic vibration was uploaded to a computer via the data acquisition board. The testing method for the amplitude is basically the same as the one described by Gavin [13]. The schematic diagram of the ultrasonic amplitude measurement of the Ti6Al4V vibrator is shown in Figure 2. The resonant frequency (*Fs*), dynamic resistance (*R*_1_), mechanical quality factor (*Qm*) and average ultrasonic amplitude of as-received Ti6Al4V alloys are 55.13 kHz, 61.36 Ω, 901.64 and 17.03 µm, respectively. The tested sample length is set as 41.25 mm.

## 3. Results and Discussion 

### 3.1. Microstructure of Ti6Al4V Alloys and Ultrasonic Resonance Parameters of Vibrators

**Figure 3:**
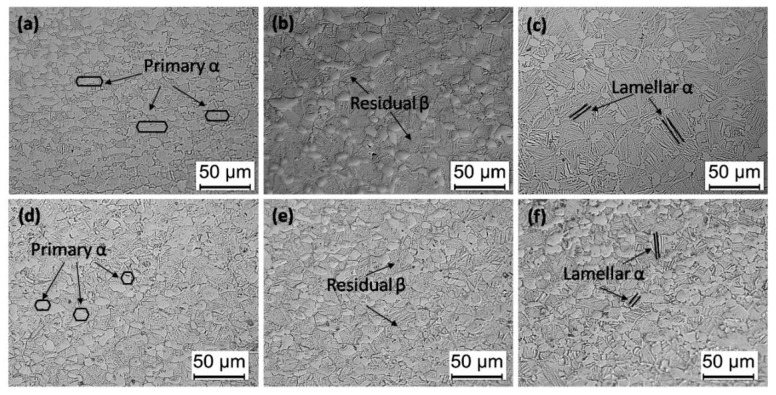
Microstructure of Ti6Al4V alloy treated by: (**a**) 920 °C × 1 h, AC, (**b**) 960 °C × 1 h, AC, and (**c**) 980 °C × 1 h, AC, (**d**) 960 °C × 1 h, AC + 600 °C × 2 h, AC, (**e**) 960 °C × 1 h, AC + 650 °C × 2 h, AC, (**f**) 960 °C × 1 h, AC + 700 °C × 2 h, AC.

**Figure 4:**
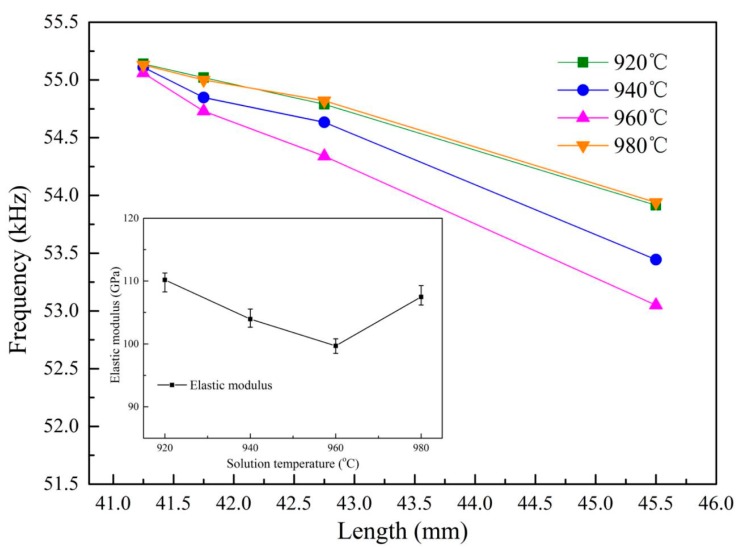
Dependence of the resonant frequency on the solid solution temperature and the sample length of Ti6Al4V alloy vibrator.

**Figure 5:**
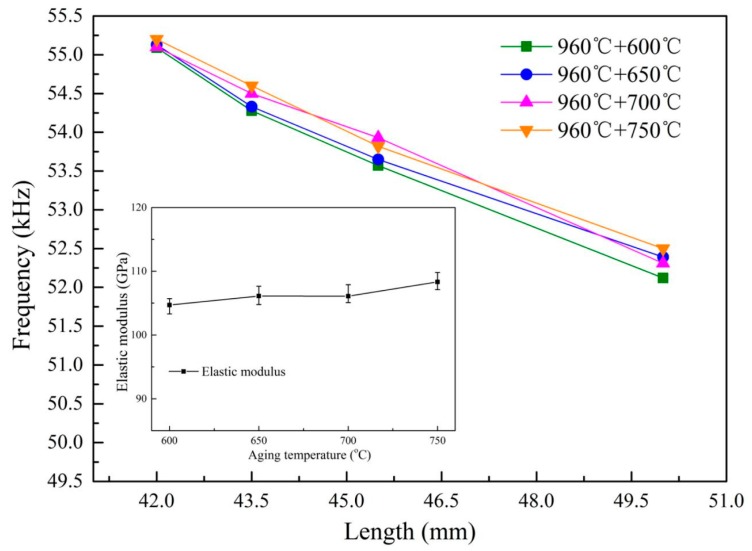
Dependence of the resonant frequency on the aging temperature and the sample length of Ti6Al4V alloy vibrator.

Figure 3 shows the microstructure of the Ti6Al4V alloys along the axial direction after different heat treatments. It is clear that the shape, content and size of the primary α, lamellar α and β phase of the alloys present great differences, respectively. The content and size of the phase grains of heat-treated alloys has been listed in detail in the first paper, and the mechanism of microstructure control has been discussed as well. Figure 4 and Figure 5 show the ultrasonic resonant frequency of the Ti6Al4V alloys with different microstructures shown in Figure 3 and the dependence of the resonant frequency on the sample length, respectively. It is clear that there is a strong dependence of the ultrasonic resonance frequency of the Ti6Al4V vibrator on the microstructure of the Ti6Al4V alloys. As shown, samples treated by solid solution treatment at 960 °C with 45.5 mm length show the lowest resonant frequency (53.05 kHz), which might be closely related to the highest residual β phase content (40.2%) of the alloys as shown in Figure 3b. It also shows that the residual β phase content slightly decreases to ~35% with increasing aging temperature. In contrast, the resonant frequency of the Ti6Al4V vibrator increases to 55.2 kHz with the sample length at 42 mm, as shown in Figure 5.

It has been shown in the first paper that the residual β phase content could be considered as the main factor affecting the elastic modulus of Ti6Al4V alloys. Additionally, the enlarged parts of Figure 4 and Figure 5 also show that the elastic modulus of the alloys is closely related to the resonant frequency of the Ti6Al4V vibrator. Therefore, the various resonant frequencies of Ti6Al4V vibrators could stem from the different elasticity of the alloys due to the different residual β phase content. It is in a good consistence with the reported results [7], in which the variation in ultrasonic parameters is due to the mismatch of the elastic modulus due to the difference in the microstructure of the two-phase titanium alloys. 

It is also noticed that the resonant frequency of the vibrators is not only related to the elastic modulus of the alloy, but also to the length of the samples, as shown in Figure 4 and Figure 5, respectively. Especially, we would like to mention that the length of the samples increases to 43.5 mm from 42 mm and the resonant frequency decreases by 1.012 kHz after they were treated by aging treatment at 600 °C. This is consistent with the reported results [15], in which the length and elasticity of the vibrator are two key parameters affecting the resonant frequency. The specific relationship between the resonant frequency and the length and elastic modulus of the sample is described by the equations as follows:

(1) c=λf

(2) $$c=\sqrt{E/\rho }$$

where *c*, *λ* and *f* are the wave speed, the wavelength and the frequency, respectively. *E* and *ρ* are the elastic modulus and the density of the alloys, respectively. Furthermore, it was proposed [15] that in order to ensure the resonance, Equation (3) as follows must be satisfied:

(3) l=nλ/2

where *l* is the length of the sample under the resonance condition and *n* = 1, 2, 3, 4, etc. Therefore, the relationship between the resonant frequency and elastic modulus and length can be derived as Equation (4):

(4) $$f=n\sqrt{E/\rho }/2l$$

It is clear that the resonant frequency is proportional to the elastic modulus and inversely proportional to the sample length. It further explains why the resonant frequency of the vibrator increases as the elastic modulus of the alloy increases, and decreases as the length of the sample increases.

**Figure 6:**
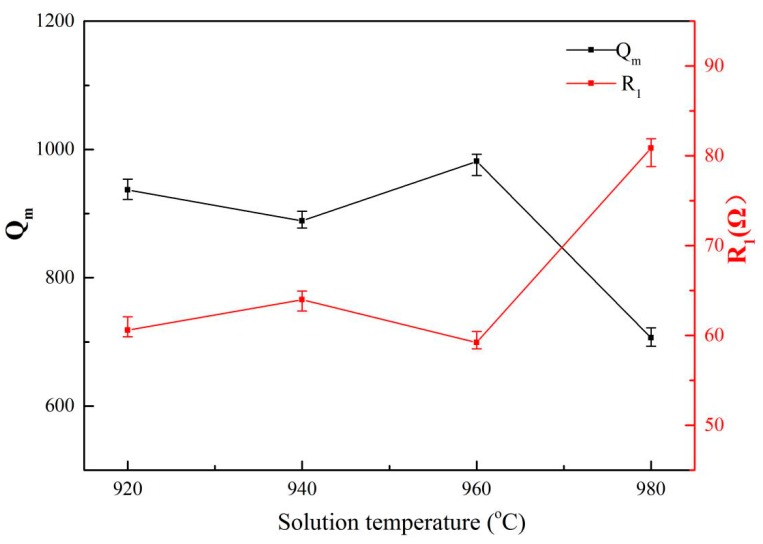
Ultrasonic resonant parameters of Ti6AlV alloy vibrator under different solution temperature.

Figure 6 shows the relationship between the mechanical quality factor (*Q_m_*) and the dynamic resistance (*R*_1_) of the Ti6Al4V vibrators after solid solution treatment. The resonant frequencies of these samples were ~55.1 ± 0.04 kHz with the length at 41.25 mm. It can be seen that the change of *R*_1_ and *Q_m_* of the vibrator have an opposite trend, again which is consistent with the reported conclusion [15] that the relationship between *Q_m_* and *R*_1_ satisfies the equation as follows:

(5) $$Q_{m}=1/R_{1}\sqrt{L_{1}/C_{1}}$$

where *L*_1_ and *C*_1_ are the dynamic inductance and dynamic capacitance of the vibrator, respectively. Where *Q_m_* denotes the mechanical energy loss during a vibration cycle. It is clear that the *Q_m_* is inversely correlated with *R*_1_ according to Equation (5).

It is shown in Figure 6 that the *Q_m_* of the Ti6Al4V vibrator does not follow a single linear relationship with increasing solution temperature, but takes 940 °C and 960 °C as turning points, and splits this trend into three regions. It is observed that when the solution temperature increases to 940 °C from 920 °C, the average *Q_m_* decreases to 888.73 from 936.8. It might be attributed to the increase in the internal friction. It was reported [16] that the mechanical loss of vibrator during a vibration cycle consists of the internal loss and surface loss. The internal loss is caused by the internal friction of the alloys. Amadori [17] further pointed out that the greater internal friction is caused by the finer grain size of the Ti6Al4V alloy. Therefore, the decrease in the *Q_m_* of the specimens’ solid solution treated at 940 °C might be ascribed to the increasing internal loss which is mainly caused by the decrease in the average grain size and content of the primary α phase, roughly 0.47 µm and 4.6%, respectively, as shown in Figure 3.

Figure 6 also shows that the *Q_m_* reached a maximum value of 981.4 as the solid solution temperature increased to 960 °C from 940 °C. This might be considered as the effect of the decrease in the surface loss of the alloys. It was pointed out [16] that the quality factor *Q_surf_* determined by the surface energy loss of cylinder samples is expressed by the following equation:

(6) $$1/Q_{surf}=4h(1/2L+1/D)\times ET\alpha^{2}/c\times \omega_{0}\tau /\left(1+{\left(\omega_{0}\tau \right)}^{2}\right)$$

where *h* is the thickness of the surface layer, *T* is the temperature, *L* and *D* are the height and diameter of the cylinder, respectively; where *E*, *α* and *c* are the elastic modulus, thermal expansion coefficient and specific heat capacity of the alloys, respectively. *ω* is the resonant angular frequency and *τ* is a constant related to the material. It is obvious that the surface loss is proportional to the elastic modulus of the alloys. Therefore, the increase in the *Q_m_* that is mentioned above could be the result of the decrease in the surface loss, which results in the 4.5% decrease in the elastic modulus of the alloys.

As shown, the *Q_m_* of the alloys rapidly decreases to 706.6 when the solution temperature increases to 980 °C from 960 °C, which can be attributed to two factors. On one hand, the internal friction of the vibrators might increase because the content and thickness of lamellar α in the alloys treated by solid solution treatment at 980 °C both increase significantly by about 24% and 95%, respectively, compared with the alloys treated by the solid solution treatment at 960 °C, as shown in Figure 3b,c. On the other hand, the residual β phase content of the alloys decreases 15% with increasing solid solution temperature, which increases the elastic modulus by about 10 GPa and subsequently causes an increase of the surface loss according to Equation (6).

**Figure 7:**
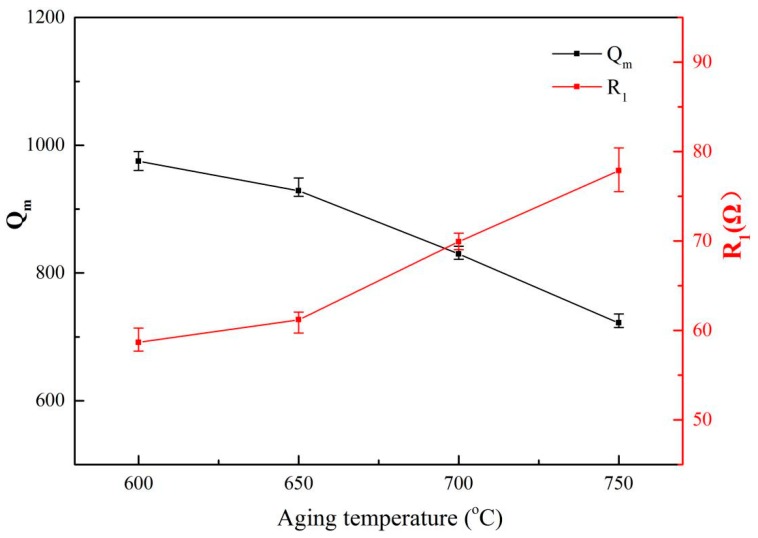
Ultrasonic resonant parameters of Ti6Al4V alloy vibrator under different aging temperature.

Figure 7 shows the relationship between the mechanical quality factor (*Q_m_*) and the dynamic resistance (*R*_1_) of the Ti6Al4V vibrators after the aging treatment. The resonant frequencies of these samples are ~55.14 ± 0.05 kHz with the length at 42 mm. Unlike the change trend of the *Q_m_* of Ti6Al4V vibrator after solid solution treatment, the *Q_m_* of vibrators after aging treatment shows a monotonous decrease with increasing aging temperature. It is obvious that the *Q_m_* decreases by 25.9% as the aging temperature increases to 750 °C from 600 °C. It has been shown in Figure 3 that the aging treatment has relatively little effect on the content of the primary α and lamellar α of the alloys. As a result, the internal friction of the vibrators during a vibration remains unchanged. Therefore, the decrease of *Q_m_* might be due to increasing surface loss, which could be caused by the increasing elastic modulus of the alloys as the aging temperature increased to 750 °C from 600 °C.

### 3.2. Microstructure and Ultrasonic Amplitude of Ti6Al4V Alloys 

**Figure 8:**
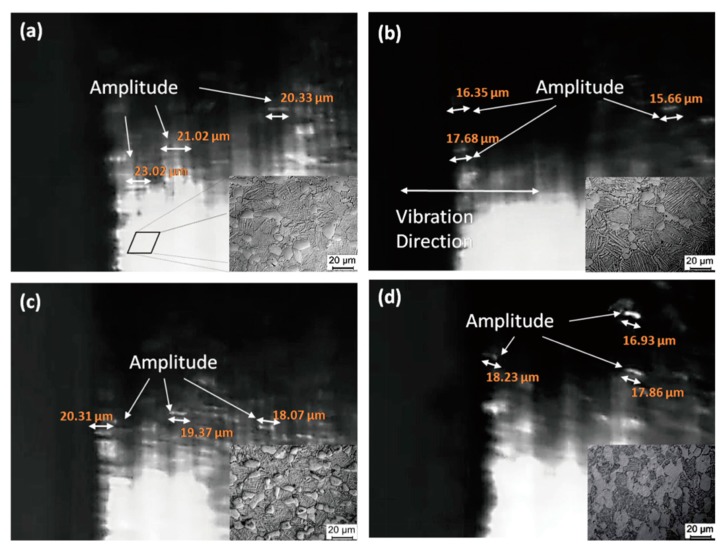
Images of the vibrating and microstructure of Ti6Al4V alloy vibrator under different heat treatment: (**a**) 960 °C × 1 h, AC; (**b**) 980 °C × 1 h, AC; (**c**) 960 °C ×1 h, AC + 600 °C × 2 h, AC; and (**d**) 960 °C × 1 h, AC + 750 °C × 2 h, AC.

Figure 8 shows the vibration images of the tip of the Ti6Al4V alloys with various microstructure at the resonance frequency of 55.5 ± 1 kHz. The results show that the microstructure can significantly affect the amplitude of the alloys. The samples treated by solid solution treatment at 960 °C with the highest residual β phase content ~40.2% show the maximum value of amplitude ~23.02 µm, as shown in Figure 8a. It then rapidly decreases to the minimum value of 15.66 µm while the residual β phase content decreases to 25.2%, as shown in Figure 8b. The amplitude of the tip of the alloys decreases slightly (~2 µm) as the aging temperature increases to 750 °C from 600 °C, which is in a good consistence with the (~5%) decrease of the residual β phase content as shown in Figure 8c,d. It has been proven that the elastic modulus of Ti6Al4V alloys is related to the residual β phase content in the present investigation. Therefore, the ultrasonic amplitude is related closely with the elastic modulus of the alloys. 

**Figure 9:**
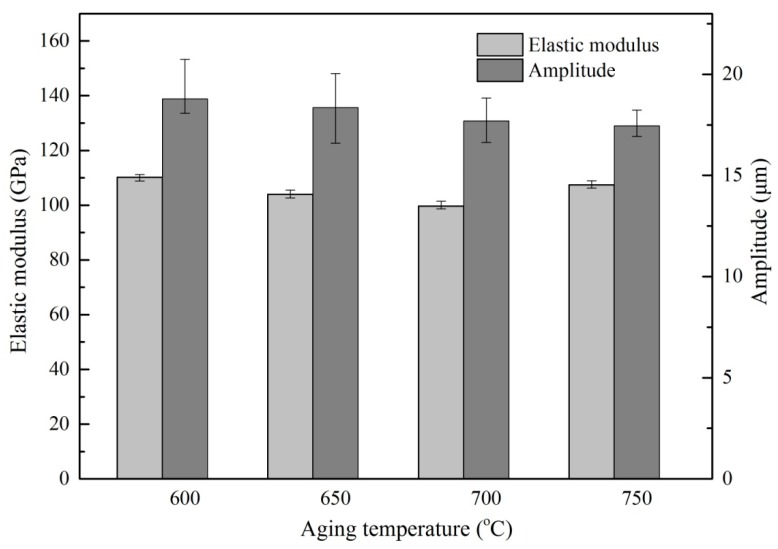
The ultrasonic amplitude and the elastic modulus of Ti6Al4V alloy specimens under different solid solution temperature.

**Figure 10:**
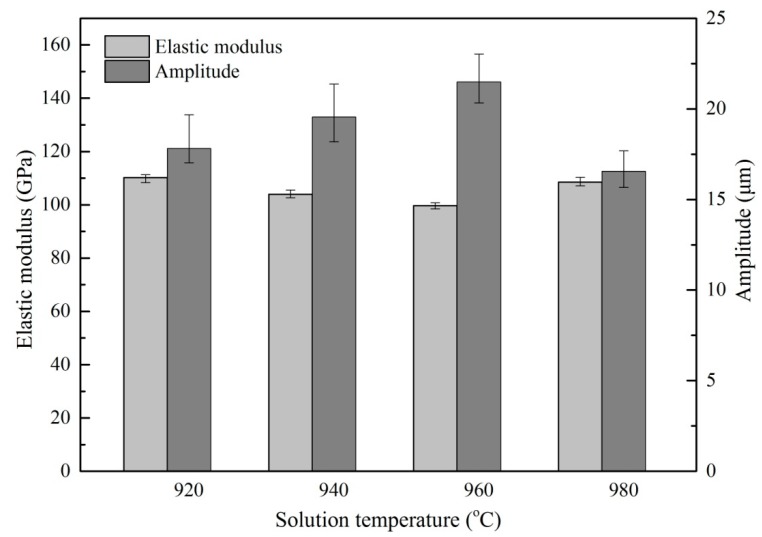
The ultrasonic amplitude and the elastic modulus of Ti6Al4V alloy specimens under different aging temperature.

Figure 9 and Figure 10 show the average ultrasonic amplitude of the tip of Ti6Al4V alloys treated by different heat treatment at the resonance frequency of 55.5 ± 1 kHz and the dependence of the elastic modulus of the alloys on the heat treatment, respectively. The results clearly show that the average amplitude is inversely correlated to the elastic modulus of the alloys. A maximum value (21.48 µm) of average amplitude is observed as the alloys are treated by solid solution treatment at 960 °C. This observation is consistent with the lowest value (99.69 GPa) of elastic modulus of the alloys, as shown in Figure 9. After aging treatment, a decrease (~10%) in average amplitude has been found, a trend that is also consistent with the increase (~7%) in the average elastic modulus of the alloys after aging treatment as shown in Figure 10.

It is well known [18,19,20] that a lower elastic modulus corresponds to a weaker binding force between atoms. The ultrasonic energy therefore enables the alloys with the lower interatomic force to move a longer distance and exhibits larger amplitude. The relationship between the amplitude and elastic modulus of the alloys was further studied in the present investigation. The displacement and amplitude are expressed as Equation (7), assuming that the harmonic vibration reaches the steady state:

(7) ξ=Acos(ωt−θ)

where *ζ* and *A* are the displacement and amplitude respectively, *ω* is the angular frequency of the driving force and *θ* indicates the phase relation between the displacement and driving force. The relationship between vibration velocity and amplitude can be derived as Equation (8):

(8) v=−Aωsin(ωt−θ)

Therefore, the expression of the maximum amplitude is:

(9) $$A_{Max}=v_{a}/\omega =v_{a}/2\pi f$$

where *v_a_* is the maximum vibration velocity, *f* is the frequency of the driving force. The maximum vibration velocity is expressed as:

(10) $$v_{a}=P_{a}/\rho_{0}c_{0}$$

where Pa is the sound pressure; *ρ*_0_, *E*_0_ are the density and elastic modulus of the sample, respectively; *c*_0_ is the wave velocity. Thus, Equation (9) can be converted to:

(11) $$A_{Max}=P_{a}/2\pi f\sqrt{\rho_{0}E_{0}}$$

Since the ultrasonic generator and transducer used in all the tests are the same, and the experimental conditions are also consistent, it can be inferred that the *P_a_* of all specimens is consistent as well as the density of the alloys. Therefore, Equation (11) indicates that the amplitude increases in accordance to an increase in the resonant frequency and the decrease in the elastic modulus of the alloys. As shown in Equation (4), the resonant frequency of the vibrator is positively correlated with the elastic modulus of the alloy. 

### 3.3. Relationship between Microstructure, Mechanical Properties, and Ultrasonic Properties of Ti6Al4V Alloys

**Figure 11:**
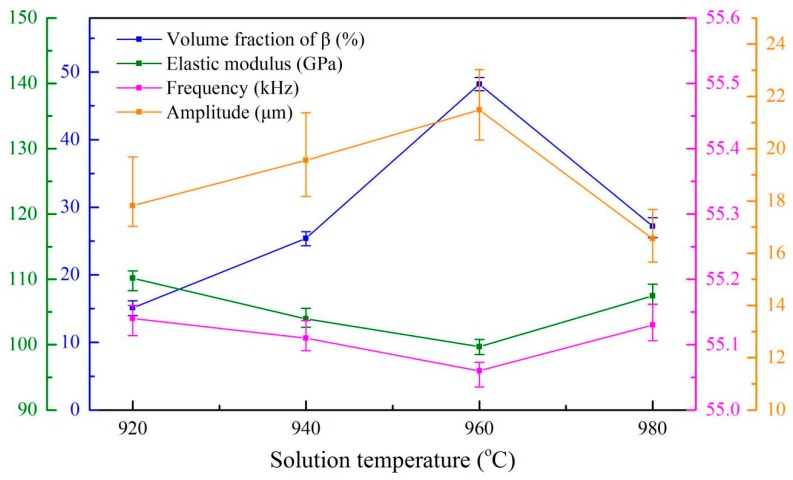
The relationship between β phase content, the elastic modulus, the ultrasonic resonant frequency and the amplitude of Ti6Al4V alloy under different solution temperature.

**Figure 12:**
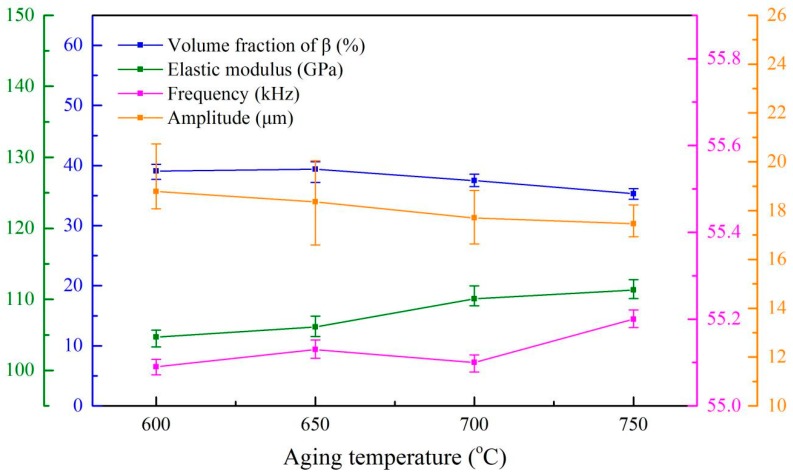
The relationship between β phase content, the elastic modulus, the ultrasonic resonant frequency and the amplitude of Ti6Al4V alloy under different aging temperature.

As shown in Figure 11 and Figure 12, a clear relationship between the microstructure, mechanical properties and ultrasonic properties of the Ti6Al4V alloys can exist in the present investigation. As expected, these results clearly show that the ultrasonic properties are strongly dependent on the elastic modulus and residual β phase content of Ti6Al4V alloys. It is observed that the residual β phase content increases (~20%) as the solid solution temperature increases to 960 °C from 920 °C as shown in Figure 11, which can explain why the elastic modulus of the alloys decreases by about 11%. It results from the lower elastic modulus of the β phase in the titanium alloys (~80 GPa), which is lower than that of the α phase (~120 GPa) [18,19,20].

It can be known from Equations (4) and (11), the elastic modulus is proportional to the resonant frequency of the vibrator and inversely proportional to the ultrasonic amplitude of the alloys, respectively. Therefore, ~11% decrease in the elastic modulus mentioned above results in having the resonant frequency of the vibrator decrease to 55.06 kHz from 55.14 kHz, while the average amplitude of the alloys increases to 21.48 µm from 17.82 µm with the sample length at 41.25 mm, as shown in Figure 11.

It has been shown that when the solid solution treatment temperature is higher than the β transus temperature (970.2 °C), the microstructure of these alloys is full of β phase and sufficiently converts into the lamellar α during the air cooling, which could result in the decrease of the residual β phase. Therefore, it is believed that the reduction (~15%) of the residual β phase causes ~9% increase in the elastic modulus of the alloys treated by solid solution treatment at 980 °C compared with the samples treated by solid solution treatment at 960 °C. 

As shown in Figure 11, this results in a 0.07 kHz increase and a 4.92 µm decrease in ultrasonic resonant frequency and amplitude of the samples, respectively, when the length was set as 41.25 mm, as shown in Figure 11. 

In contrast, Figure 12 shows that the residual β phase content, the elastic modulus and the ultrasonic properties (resonant frequency and amplitude) of the alloys with 42 mm in length endured different aging treatment. It is clear that a higher resonant frequency (~0.07 kHz) can be found in these samples than those treated by solid solution treatment at 960 °C. It is believed that it might be due to a longer sample and a higher elastic modulus of these alloys as induced by Equation (4). We would like to stress that a ~6 GPa increase in the elastic modulus would result in an average 3.4 µm decrease in the amplitude of these specimens.

## 4. Conclusions

The relationship between the ultrasonic properties, the elastic modulus and the β phase content of the Ti6Al4V alloys has been clarified. The reduction of the residual β phase content lowers the elastic modulus of the alloys that subsequently results in a decrease in the resonant frequencies and an increase in the average amplitude of the alloys. The alloys treated by solid solution treatment at 960 °C present a bimodal structure with certain β phase content (~40%) and a lamellar α thickness of ≤0.9 µm and exhibit excellent ultrasonic and mechanical properties. The resonant frequency is the lowest (55.06 kHz). The average amplitude is the largest (21.48 µm), a ~26.5% increase compared with that of as-received alloys at a length of 41.25 mm. The present investigation could be helpful to guide the design of Ti6Al4V ultrasonic scalpels and their improved efficiency in ultrasonic surgery applications.
